# Association of *MEF2A *Gene Polymorphisms With Coronary Artery Disease

**DOI:** 10.5812/ircmj.13533

**Published:** 2014-08-05

**Authors:** Ali Mohammad Foroughmand, Zahra Shahbazi, Hamid Galehdari, Mahdi Purmahdi Borujeni, Parvane Dinarvand, Khadije Golabgirkhademi

**Affiliations:** 1 Faculty of Science, Department of Genetics, Shahid Chamran University of Ahvaz, Ahvaz, IR Iran; 2 Faculty of Veterinary Medicine, Department of Food Hygiene, Shahid Chamran University of Ahvaz, Ahvaz, IR Iran

**Keywords:** Coronary Artery Disease, Polymorphism, Single Nucleotide, Polymorphism, Restriction Fragment Length

## Abstract

**Background:**

Coronary Artery Disease (CAD) is the most common cause of death worldwide. MEF2A directly regulates target genes in the process of muscle development. This gene product is a transcription factor. MEF2A protein in homodimer or heterodimer forms binds to A/T-rich *cis* elements with conserved sequence in promoter, regulator, and enhancer of many genes, which are determining in evolution and development of skeletal, heart, and smooth muscle cells, especially endothelial cells. In fact, this protein maximizes the activity of these elements.

**Objectives:**

The two *MEF2A* gene polymorphisms that were proposed to have an association with CAD are rs34851361 (A/G) and rs325400 (T/G) SNPs. This study aimed to examine these associations.

**Patients and Methods:**

This study was a molecular case-control study. Blood samples were collected from 300 patients with CAD and 150 healthy people from Golestan and Imam Khomeini Hospitals, Ahvaz, Iran. In both groups, angiography had confirmed the presence or lack of stenosis. Association of rs34851361 and rs325400 with CAD was evaluated by PCR and then restriction fragment length polymorphism (RFLP) analysis was performed.

**Results:**

Chi square test showed no association between rs34851361 SNP and CAD (χ^2^ = 3.59, df = 2, and P = 0.16); however, there was an association between rs325400 SNP and CAD (χ^2^ = 24.77, df = 2, and P < 0.001). A/T haplotype showed association with CAD and G/G and G/T showed protective effect against CAD.

**Conclusions:**

The results of this study show that rs325400 polymorphism is in association with CAD; meanwhile, none of the rs34851361 genotypes was associated with CAD.

## 1. Background

Coronary artery disease (CAD) is most commonly caused by atherosclerotic occlusion of the coronary arteries. Atherosclerosis can involve different blood vessels and when it involves the coronary arteries, it leads to CAD. The major cause of CAD is atherosclerosis. Atherosclerosis process begins with the disruption of endothelial function due to the accumulation of lipoprotein droplets in the intima of the coronary vessels. Factors that are believed to impair endothelial function and lead to CAD include atherosclerosis, dyslipidemias, diabetes mellitus, hypertension, smoking (passive or active), and obesity. When the atherosclerotic process prevents blood flow through the coronary artery, myocardial infarction occurs (1, 2). Recent research has shown that inflammation plays a key role in CAD and other manifestations of atherosclerosis (2, 3). Endothelial dysfunction may be the earliest stage of coronary atherosclerosis (4). Atherosclerosis process includes complex events and each one involves a specific biological pathways and different genes. CAD is a complex disease caused by interactions among multiple genetic and environmental factors. Both of these factors reduce the quality of life and greatly increase the mortality rate. Multiple approaches have been developed to understand the genetic basis of these complex disorders (5-7). Half of all deaths in the developed countries and a quarter of deaths in the developing countries are due to cardiovascular disease (1). CAD is the major cause of morbidity and mortality in the majority of industrialized societies (8, 9). In Iran, it accounts for approximately 50% of all deaths per year (4). According to global and regional projections of mortality and burden of disease, CAD will remain the main cause of death for the next 20 years (10).

Wang et al. published a paper in science journal in which they first introduced myogenic enhancer transcription factor 2a (*MEF2A*) gene as the cause of CAD (11). Thereafter, many studies were conducted on this gene and its effect on CAD. Some studies negated the role of *MEF2A* in CAD while others confirmed its influential role (12-16). The outcome of this gene is a transcription factor that binds to DNA. Homodimer or heterodimer forms of MEF2A protein bind to A/T-rich *cis* elements that have (C/T) TA (A/T) 4 TA (G/A) conserved sequence in promoter, regulator, and enhancer of many genes, which are effective in evolution and development of skeletal, heart, and smooth muscle cells, especially endothelial cells, and maximizes their activity (16-18). 

In 2010, Elhawari et al. studied the association of *MEF2A* gene single-nucleotide polymorphisms (SNP), namely, rs325400 G > T and rs34851361 A > G, with CAD and demonstrated borderline association with rs34851361 and no association with rs325400. These two SNPs are in 11th exon and are silence; however, rs325400 codes for glycine and rs34851361 codes for proline (19, 20).

## 2. Objectives

This study aimed to evaluated the association of CAD with two *MEF2A* gene SNPs, namely, rs325400 G > T and rs34851361 A > G. 

## 3. Patients and Methods

**Figure 1.**
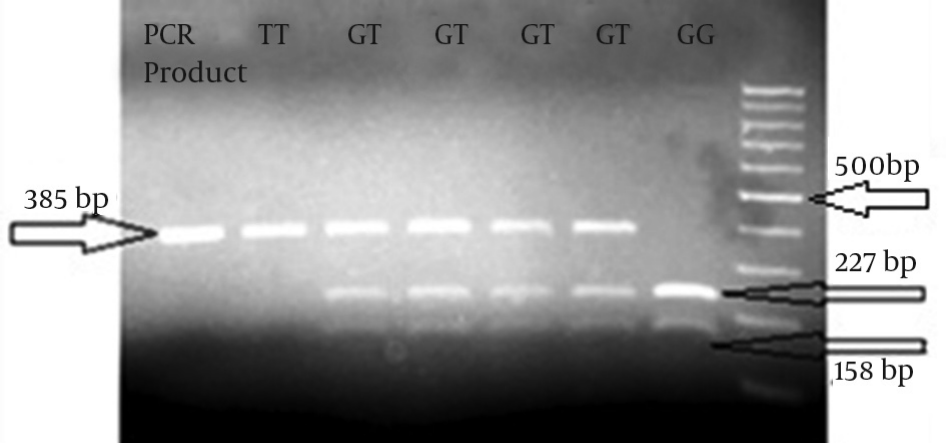
Restriction Fragment Length Polymorphism for rs325400

**Figure 2.**
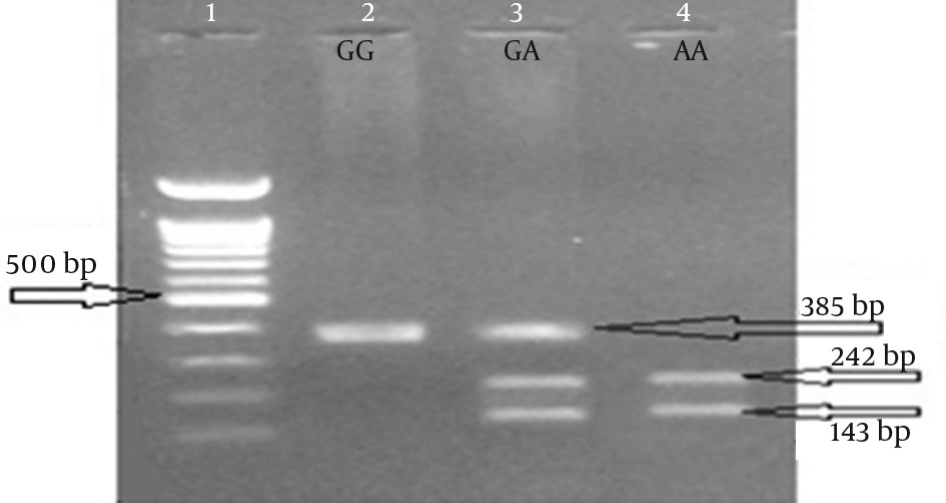
Restriction Fragment Length Polymorphism for rs34851361

in this case-control study, samples were collected from the people who were referred to Angiography Department of Golestan and Imam Khomeini Hospital, Ahvaz, Iran, which are governmental university general hospitals. Sampling was done under the supervision of a specialist. Participants were informed about our study and they signed out an informed consent form. Patients filled in and signed questionnaire and testimonial at the time of admission. All data related to the samples are presented in Table 1. The number of participants in the case and control groups was 300 and 150, respectively. A 5 mL blood sample from each patient was decanted into test tubes containing 0.5 mL of 0.5 M EDTA as anticoagulant. Then we extracted DNA with Diatom DNA prep 100 kit (IsoGene Company). The two studied SNPs are 86 nucleotides away from each other and can be amplified by one pair of PCR primers. PCR product is 385 nucleotides in length. Next, we did restriction fragment length polymorphism (RFLP) with two restriction endonucleases (from Fermentase Company) named *HhaI* for rs325400 G > T (Figure 1) and *MfeI* (*MunI*) for rs34851361 A > G (Figure 2). Digestion product could be detected with agarose-gel electrophoresis. Statistical analysis was performed by SPSS version 16.0 (SPSS Inc., Chicago, IL, USA). Analysis of data was performed by Chi square test, Fisher’s exact test, and Logistic regression.

**Table 1.** Demographic Characteristics of the Participants in Each Group ^a^

Variables	Cases	Controls
**Age, y **	53.42 ± 7.3	60.2 ± 7.94
**Sex**		
Male	176	64
Female	124	86
**Ethnicity**		
Arabs	138	74
Non-Arabs	158	74
Missing Data	4	2
**Blood Pressure**		
Affected	132	52
Not Affected	166	97
Missing Data	2	1
**Diabetes Mellitus**		
Affected	103	43
Not Affected	185	106
Missing Data	12	1
**Blood Lipids**		
Affected	143	49
Not Affected	156	100
Missing Data	1	1

^a^Data are presented as mean ± SD or frequency.

## 4. Results

The rs34851361 polymorphism was not significantly associated with CAD (χ^2^ = 3.59, df = 2, and P = 0.16); however, rs325400 had a significant association with CAD (χ^2^ = 24.77, df = 2, and P < 0.001). In these two SNPs, there were no significant differences in genotype frequencies between men and women in the case or in the control groups. The results of sex calculation for rs325400 and rs34851361 were as follows: χ^2^ = 2.28, df = 2, and P = 0.32; and χ^2^ = 0.55, df = 2, and P = 0.76, respectively. The results of further analysis showed no significant difference in genotype frequencies between Arab and Non-Arab ethnicities in both of the polymorphisms. The results of ethnicity calculations for rs325400 and rs34851361 were as follows χ^2^ = 0.065, df = 2, and P = 0.97; and χ^2^ = 2.1, df = 2, and P = 0.35, respectively. We analyzed these two SNPs in haplotypes and A/T haplotype showed association with CAD development and G/G and G/T haplotypes showed protective effect against CAD (see Table 2). The results of calculations related to the association between CAD and some proposed factors such as diabetes mellitus, high blood pressure, high blood lipids, and sex are shown in Table 3.

**Table 2.** Statistical Analysis for Haplotypes ^a^

Rs34581361	Rs325400	Haplotype	χ^2^	df	P Value	OR	95% CI for OR
**A**	T	AT	12.24	1	< 0.001	2.15	1.41-3.27
**G**	T	GT	15.12	1	< 0.001	0.32	0.18-0.57
**G**	G	GG	39.5	1	< 0.001	0.096	0.04-0.22

^a^Abbreviations: OR, odds ratio; CI, confidence interval; and df, degree of freedom.

**Table 3.** Association of Coronary Artery Disease and Some Proposed Factors ^a^

Factors parameter	High Blood Lipids	Ethnicity	Diabetes Mellitus	Blood Pressure	Gender
**OR**	1.87	1.14	1.37	1.48	1.91
**OR explanation**	People with high blood lipids are 1.87 times more likely to have CAD than people without it are.	Non-Arabs are 1.14 times more likely to have CAD than Arabs are.	People with diabetes are 1.37 times more likely to have CAD than people without it are.	People with high blood pressure are 1.48 times more likely to have CAD than people without it are.	Males are 1.91 times more likely to have CAD than females are.
**95% CI**	1.24-2.82	0.77-1.69	0.89-2.11	0.99-2.23	1.28-2.84
**P value**	0.003	0.5	0.115	0.058	0.01
**χ** ^**2**^	8.46	6.73	1.81	3.38	9.65
**df**	1	3	1	1	1
**P value**	0.04	0.081	0.18	0.07	0.002
**Description**	Association between high blood lipids and CAD is significant.	Association between ethnicity and CAD is not significant.	Association between diabetes mellitus and CAD is not significant.	Association between blood pressure and CAD is not significant.	Association between sex and CAD is not significant.

^a^ Abbreviations: CAD, coronary artery disease; OR, odds ratio; CI, confidence interval; and df, degree of freedom.

## 5. Discussion

The purpose of this study was to examine the association of two *MEF2A* gene SNPs, namely, rs325400 and rs34851361, and their three genotypes with CAD in Ahvaz population. Diagnosis of CAD was based on the angiographic studies. As a limitation, of angiography only reports the presence or absence of atherosclerosis and vascular occlusion. CAD occurs in older ages; thus, it is possible for control group to be affected in the future. To minimize this effect, only those over 48 were studied among the individuals with normal angiographic result. One of the difficulties of studying CAD is in finding matched patient and control groups in terms of age. Environmental effects become greater with advancing age. Therefore, many elderly people were excluded because it was not clear whether the cause of disease was genetic or environmental factors. Our inclusion and exclusion criteria were sex and age and in both groups, angiography confirmed the presence or absence of stenosis and we did not use other diagnostic methods because angiography has the highest accuracy among the available methods. The number of male and female participants was approximately equal. CAD is a multifactorial disease caused by interactions between multiple genetic and environmental factors. Then, with advancing age, CAD may be the result of environmental factors. We aimed to study the effect of genetic factors and we had to exclude environmental factors as much as possible. For this purpose, the cases and controls were selected from those who were younger than 65 years and older than 48 years, respectively. Cases included those who had afflicted by CAD in younger ages and had high disease susceptibility; controls were those without CAD in spite of aging and therefore, might have the ability to resist the disease. With these criteria, approximately 450 samples from 1471 patients met the eligibility criteria and were recruited in this study. The measurement methods of clinical variables included fasting blood sugar test for diabetes, LDL, HDL, and VLDL tests for lipid profile, and blood pressure test at the time of admission, a history of hypertension, and antihypertensive drug use for hypertension. With regard to all these factors, doctors and specialists classified the samples into afflicted and non-afflicted groups on the basis of medical criteria. In this study, the maximum age of the patients was 65 years. This age restriction was implemented to minimize the effect of environmental factors for cases and to maximize its effect for controls. Having this disease in younger ages shows higher genetic predisposition for CAD and minimal effect of environmental factors. Moreover, being healthy in older ages shows low genetic predisposition for this disease that confronts probable inappropriate environmental conditions.

Another difficulty in the study of this disease was matching participants for sex. In general, more men than women are diagnosed with CAD. This could be due to the protective role of estrogen in women. In this study, the chances of developing CAD was estimated and found to be 1.91 times more for men than for women; this difference was statistically significant. A non-modifiable risk factor for CAD is male gender. Among the modifiable risk factors, hypertension and diabetes have shown no significant association with CAD. Lipid is another factor that has shown a statistically significant association with CAD. In analysis of the genotypes of the samples, in rs34851361 polymorphism, frequency of AA genotype was found to be higher in patients in comparison with healthy people while the frequency of the other two genotypes, i.e. AG and GG, was higher in healthy people. The frequency of A and G alleles was higher respectively in patients and the healthy group. Overall, this polymorphism was not significantly associated with CAD. In rs325400 polymorphism, frequency of TT genotype was higher in patients and the frequency of TG and GG genotypes was higher in healthy people. The frequency of T allele in patients and G allele in healthy people was higher. Overall, this polymorphism was significantly associated with CAD.

Due to ethnic variety and differences in Ahvaz, the chances of disease and genotype frequency was determined based on ethnicity (Arab, non-Arab). Although the chance of non-Arab ethnicity for CAD was higher than Arab ethnicity, this difference was not statistically significant. Regarding the studied two SNPs, no significant difference in genotype frequencies was found between Arabs and non-Arabs. Moreover, analysis of genotype frequency did not reveal any significant difference between the two sexes.

This study has some weaknesses and strengths that are worth of consideration. In association studies, the sample size is an important factor. This was a shortcoming of the study and it was due to large number of eligibility criteria that should be met in our sampling. In addition, measurement of clinical factors was inaccurate in spite of using the most accurate method, which is due to intrinsic properties of these quantities. One of the strengths of this study was the sampling method as angiography has high accuracy. Another strength of the study was the used molecular methods in this study; PCR and RFLP have high validation. In addition, we tried to study some other risk factors. The association of CAD with blood pressure and diabetes mellitus was insignificant, which might be due to the small sample size. Nevertheless, these risk factors have increased the likelihood of CAD (Table 3). Another reason might be the uncertainty of measurement methods. Some studies have rejected the role of *MEF2A* gene in CAD (12) and some others have confirmed it (11, 13, 16). Among the second group, these two SNPs were also considered a study by Elhawaria et al. in Saudi Arabia. In Elhawari et al. study, Rs325400 did not show a significant association with CAD; in contrast, rs34851361 showed boundary association with CAD (19). In our study, rs325400 had a significant association with CAD and none of the rs34851361 genotypes had association with CAD.

The results of this study showed that TT genotype of rs325400 polymorphism had a significant association with CAD, and ethnicity and sex had no effect on genotype frequency of this polymorphism. Meanwhile, none of the rs34851361 genotypes was associated with CAD. Ethnicity and sex had also no effect on genotype frequency of this polymorphism.
